# HRM models of online labor platforms: Strategies of market and corporate logics

**DOI:** 10.3389/fsoc.2022.980301

**Published:** 2023-01-06

**Authors:** Jere Immonen

**Affiliations:** ^1^Faculty of Humanities and Social Sciences, University of Jyväskylä, Jyväskylä, Finland; ^2^Finnish Institute of Occupational Health, Helsinki, Finland

**Keywords:** online labor platforms, institutional logics, human resource (HR) management, market logic, classification, corporation logic, platform work, governance principles

## Abstract

Studies on online labor platforms (OLPs) have revealed that OLPs can have extensive managerial control over independent workers, which affects their autonomy and precariousness. The permeability of the management makes some OLPs' roles as neutral intermediaries in labor exchanges questionable. While there are several platform work studies on the effects of human resource management (HRM) activities, earlier studies have focused more on certain types of OLP companies. Earlier OLP classifications did not make systematic distinctions between HRM activities either. This paper offers a classification to view how HRM activities manifest in OLPs. The study utilizes terms of service and webpage data from 46 multinational and Finland-based OLPs. Based on these data, OLPs have been classified into six models with five governance principles and institutional logic. The study uses the idea of institutional complexity and claims that OLPs balance their operations between the complexity of two institutional logics, market, and corporation, by using varying strategies with their HRM activities. Differently managed OLPs are also often marketed to different worker groups. This indicates that workers' levels and quality of autonomy differ between OLPs. Hence, could be expected that platform workers' expectations toward OLPs, perceptions of fairness, and experiences of wellbeing may be influenced by the HRM activities in which they engage. The results contribute to the ongoing discussions of power asymmetries between OLPs and platform workers, and thus OLPs' roles as either marketplaces or hierarchical corporations. Formed models can be utilized to enrich studies on key issues of platform workers' autonomy, precariousness, and experiences in different types of OLPs.

## Introduction

Businesses built on the intermediation of decentralized exchanges among peers through digital platforms have extensively found their way into various sectors of economic life in the 2010s, creating new ways of working and earning (Acquier et al., 2017; Dieuaide and Azaïs, 2020; Vallas and Schor, 2020; International Labour Organization, 2021). Even though they are often referred to as new, many platform-based companies are already a decade old, and the diversification of services on digital platforms has been ongoing for several years (Vallas and Schor, 2020). This diversity is visible on platforms where mediated services are labor-intensive, often referred to as “online labor platforms” (OLPs) (e.g., Kuhn and Maleki, 2017; International Labour Organization, 2021; Keegan and Meijerink, 2021).

Previous classifications have drawn the variation of OLP models. OLPs have been classified based on the type of work, required skills, online- or on-location performance, and work allocation types and sectors (e.g., Eurofound, 2018; Kenney et al., 2020; International Labour Organization, 2021). However, these classifications have yet to reveal the variety of OLPs' human resource management (HRM) activities. A review of HRM activities is essential when evaluating relationships between OLPs and workers and possible power asymmetries between them. The power relations question has been represented in studies and debates on labor control and employment statuses in platform work. Found power asymmetry structures have caused contradictions in OLP companies' legitimacy and legality and questions of whether OLPs should be considered employers of workers rather than neutral intermediates or marketplaces (De Stefano, 2015; Dieuaide and Azaïs, 2020; Schor et al., 2020; Connelly et al., 2021; International Labour Organization, 2021).

OLPs have a wide range of different HRM activities in their operations. Studies have mainly focused on global OLP companies like Uber, Foodora, and Amazon Mechanical Turk and have aimed to understand the nature of these activities and their individual-level effects on workers' insecurities, flexibility, and autonomy (e.g., Ivanova et al., 2018; Lehdonvirta, 2018; Peticca-Harris et al., 2020; Schor et al., 2020; Wiener et al., 2021; Wood, 2021), as well as societal effects on labor markets and employment security (e.g., Schor et al., 2020; Stewart et al., 2020). It is worth noting that earlier studies on platform work have paid far too little attention to OLPs in which work processes and work mediation are managed at lower intensities (Schor et al., 2020). It is, however, noticeable that HRM activities can translate into different elements of work depending on OLPs' businesses and what they consider strategically essential for optimizing their operations (Vallas and Schor, 2020; Keegan and Meijerink, 2021; Schußler et al., 2021) HRM activities may also affect workers' experiences of wellbeing and fairness (Seppänen et al., 2023).

This study and its classification aim to indicate the heterogeneous nature of OLP companies' HRM better than earlier classifications have done. The pursuit is to create concepts to analyze what HRM activity variations OLPs have in their operations. Debates on platform work often emphasize the challenges and possibilities of the phenomenon through certain OLP companies without considering that platform work is not a single entity but consists of different management solutions and ways of operating in various industries and types of work. This classification provides tools to recognize differences in OLPs' HRM activities. It enables further analysis of what forms of platform work are becoming more common and what types of worker-related challenges and opportunities are associated with differently managed OLPs. The questions of power relations between OLPs and workers differ based on OLPs' HRM activities. This classification helps outline the HRM models in which issues of power asymmetries, insecurities, and autonomy may be the most significant and those in which they are less present. It will also contribute to the debate on whether OLPs are marketplaces, hierarchical organizations, or something between them. The current situation, where regulations and rules for platform work are being outlined (e.g., European commission, 2021; Hießl, 2022), increases the importance of studying the phenomenon with new theories and data.

This paper's analysis subjects are multinational and Finland-based OLPs (46 OLPs), whose HRM activities are analyzed with the theory of five governance principles. Principles consist of (1) deciding on memberships, (2) governing rules, (3) monitoring rule compliance, (4) sanctioning non-compliances, and (5) establishing a hierarchy (Ahrne et al., 2015). In addition to principles, I will also analyze OLPs' employment relations with workers, suggesting whether workers operate in OLPs as entrepreneurs or with provisional or permanent employment relations.

How OLPs manage governance principles will be viewed through the lens of institutional logic theory. In work by Frenken et al. (2020) and Keegan and Meijerink (2021), OLPs have been seen to face the complexity of the market and corporate logic and respond to the complexity with different HRM activities. The presumption is that HRM activities reflect OLPs' responses to logic complexity and the centrality of either of these logics. The paper contributes to the theorization of Frenken et al. (2020) and uses the HRM approach from Keegan and Meijerink (2021) and Meijerink et al. (2021a). Using earlier studies as a foundation, this paper claims that by reviewing logic complexities through HRM activities, we can recognize different OLP models and relations between OLPs and platform workers. This information can be used to distinguish between OLPs' HRM models and classify them. The research question of this paper is as follows: *What models do online labor platform companies' HRM activities display?* With this question, I will look at what kinds of HRM models can be recognized based on the saliencies of market and corporation logic indicated by governance principles.

Earlier studies on OLPs' control schemes and institutional logic have focused on legislatively challenging cases (Frenken et al., 2020) and certain industries and types of platform work (Keegan and Meijerink, 2021; Meijerink et al., 2021b). This study takes a wider sample of different OLP companies and aims to show variations of HRM models and OLP companies' response strategies to market and corporation logic complexities. The study also responds to the need for more comparative analyses of different types of OLPs (Keegan and Meijerink, 2021). It tests whether an institutional logic approach is generalizable to different OLPs and types of platform work (e.g., Meijerink et al., 2021a).

In the study, I have created six generalized models based on HRM's response to market and corporation logic complexities: (1) free marketplaces, (2) managed marketplaces, (3) temporary employment platforms, (4) managed networks, (5) managed work processes, and (6) managed like corporations. The worker groups to which OLPs advertise their services as working opportunities have also been viewed through the models. This gives a tentative idea of what HRM activities are common in what types of work and to what groups of workers they apply. This information and these models will offer instruments to deepen future studies on workers' experience, power relations, and legitimacy questions in differently managed OLPs. The following section will present a definition of OLPs and earlier classifications from new perspectives.

## Earlier classifications and research on OLPs

Digital online labor platforms are for-profit companies that serve as digital intermediaries for temporary, paid work tasks (Kuhn and Maleki, 2017; Eurofound, 2018; Kovalainen et al., 2020). OLPs do not usually serve as service providers but rather build their businesses on enabling interactions and labor exchanges between parties (Kuhn and Maleki, 2017; Kovalainen et al., 2020; International Labour Organization, 2021; Meijerink et al., 2021b; Wood, 2021). There are various types of OLPs for mediating different kinds of work, and OLPs have been classified in many ways, usually based on work characteristics. A commonly used distinction is whether work mediated through OLPs is done online or offline (Eurofound, 2018; Kenney et al., 2020; Vallas and Schor, 2020; International Labour Organization, 2021; Meijerink et al., 2021a). In addition, ILO, among many others, distinguishes OLPs based on the sector, for example, delivery, care services, or medical consultation (International Labour Organization, 2021). Another commonly used factor in classification is the content of mediated work and required skills (Eurofound, 2018; Howcroft and Bergwall-Kåreborn, 2018; Vallas and Schor, 2020; International Labour Organization, 2021).

In Eurofound's classification, one essential element is the party that determines work allocation and the selection of workers. This separates OLPs that offer tasks directly to workers from OLPs where matching is based on free competition between workers (Eurofound, 2018). Work allocation management is one central HRM activity that OLPs often use, but it is not the only one. Workers in many OLPs are subjects of a range of HRM activities such as recruitment, appraisal, task allocation, compensation, and job design (Kuhn and Maleki, 2017; Connelly et al., 2021; Keegan and Meijerink, 2021; Meijerink et al., 2021b; Waldkirch et al., 2021). Earlier classifications have not made comprehensive distinctions between OLPs based on HRM activities, although some classifications have focused on certain dimensions of HRM, like in the case of Eurofound. There have been approaches to creating classifications of control mechanisms for digital platforms, but they have not directly focused on OLPs and labor intermediation (e.g., Maffie, 2020).

While HRM activities have yet to be comprehensively present in OLP classifications, they have been widely displayed in studies on platform work. Previous research has established that OLPs can have wide managerial control over work processes and self-employed workers, which distorts the boundaries between employment and self-employment and the roles of platform operators (Kovalainen et al., 2020; Schor et al., 2020). This contradiction between employment status and management activities has been widely featured in sociological and HRM studies. HRM studies on platform work have mainly focused on the interrelation between workers and OLPs, especially on algorithmic management and its effects on worker autonomy (e.g., Fieseler et al., 2017; Kuhn and Maleki, 2017; Duggan et al., 2019; Bucher et al., 2021; Schußler et al., 2021; Wiener et al., 2021). Sociological studies have also emphasized that algorithmic management has created precarious and unpredictable working environments for independent platform workers and promoted an erosion of employment security (e.g., De Stefano, 2015; Van Doorn, 2017; Dieuaide and Azaïs, 2020; Kahancová et al., 2020; Krzywdzinski and Gerber, 2020; Peticca-Harris et al., 2020; Schor et al., 2020).

While earlier studies have identified the versatility of HRM activities in OLPs, they have yet to make distinctions between HRM models and how activities are manifested in different OLPs. Vallas and Schor (2020) view OLPs' managerial control as a sum of strategic solutions. They claim that based on their businesses, OLPs choose to control certain important functions, but they also decentralize control of selected functions. By doing so, they transfer responsibility to platform users and trust the disciplinary power of labor markets while simultaneously centralizing their own power with carefully selected management solutions (Vallas and Schor, 2020). This indicates that OLPs' HRM activities are not always all-encompassing and extensive but vary based on the OLP companies and what they want to achieve with their operations. Therefore, some OLPs may appear as less-managed marketplaces, while others may resemble more hierarchical organizations.

As previously indicated, this complexity and diversity of HRM activities have yet to be comprehensively viewed in earlier classifications. One of the reasons for this may be that research often leads to the acknowledgment of OLPs' various strategies with their HRM activities (Eurofound, 2019; Schußler et al., 2021). While this also applies to this study, I found it possible to identify general similarities and differences between models of different OLPs based on their HRM. Next, I will introduce the theories of five governance principles and institutional logic that will be utilized to outline OLPs' HRM models and the complexity between OLPs' roles as marketplaces or hierarchical corporations.

## Governance principles and institutional logics

Standard organization theory sees organizations fundamentally as formal decisions of social orders with their own rules, expectations, and direction of activities (Ahrne and Brunsson, 2011). In the definition by Ahrne and Brunsson, these formal or “complete” organizations contain five governance principles that keep interaction predictable and continuous and are defined and managed by the organizations themselves: (1) membership and who can join, (2) organizations' rules, (3) ways to monitor work and members, (4) sanctions for non-compliance, and (5) hierarchies and the positions of members. Organizations adopt different strategies and HRM activities to manage these five principles and may not necessarily have an interest in or possibility of managing them all. Organizations that manage only some of the principles are referred to as “partial” organizations (Ahrne and Brunsson, 2011; Ahrne et al., 2015).

For this study, governance principles will offer theory and concepts to outline the dimensions to which HRM activities of OLPs can be directed. This enables us to review the nature of OLP companies as marketplaces or hierarchical Organizations. Markets and organizations are both fundamentally decisions of social interactions, with their own direction of operations, but often in market environments, governance principles are achieved in ways other than internally managed procedures (Ahrne and Brunsson, 2011). Historically, the decisions of the five governance principles in market environments have been delegated to different institutions, but digital platforms can manage them all (Kirchner and Schüßler, 2018). Thus, their nature as either hierarchical organizations or marketplaces, or complete or partial organizations, is often difficult to outline.

In HRM research, the above described complexity has been theorized with institutional logics theory (e.g., Frenken et al., 2020; Keegan and Meijerink, 2021; Meijerink et al., 2021b). Institutional logic is “*the socially constructed, historical pattern of material practices, assumptions, values, beliefs, and rules by which individuals produce and reproduce their material subsistence, organize time and space, and provide meaning to their social reality”* (Thornton and Ocasio, 1999, p. 804). The institutional logic theory sees people, organizations, and other communities entwined with multiple intersecting institutions and their expectations (Thornton et al., 2012). Organizations' strategies to respond to this tension of different institutional logics and modify their practices under this “institutional complexity” make their operations heterogeneous (Greenwood et al., 2011).

Institutional logics are pre-created templates of ideals for institutional orders (Thornton et al., 2012). Similar to Weber's interpretive framework, ideals are a set of features of given institutions but do not necessarily represent reality. Rather, ideals are deliberately highlighted features to make central elements of the investigated subject visible and intelligible (Neves and Mead, 2018). Ideals offer a framework to compare empirically noticeable aspects of the organization and conceptualize them (Thornton et al., 2012). While they have been criticized for arbitrary formulations and having more classificatory than explicatory natures (Neves and Mead, 2018), ideals are useful when outlining the complexities that OLPs have with traditional working life and marketplace practices such as HRM activities.

In earlier studies, digital platforms, including OLPs, have faced the complexity of many intersecting institutional logics. These include state logic, like laws and regulations, and the professional logic of people and their will to promote their own competence (Frenken et al., 2020; Keegan and Meijerink, 2021). The complexity that has challenged OLPs' legitimacy the most occurs between market and corporate logic. OLPs can have both managerial actions to control workers in various ways and means, but at the same time, they can aim to be marketplaces for self-employed workers to compete freely (Kuhn and Maleki, 2017; Frenken et al., 2020; Keegan and Meijerink, 2021; Meijerink et al., 2021b).

In the market logic ideal, legitimization comes through free profit-making and unregulated competition, in which operators aim to maximize their own profit and gain competitive status in market environments. Corporation logic authorizes market share and revenue growth through the coordination and control of workers. Workers' benefits are defined by their position in the organization's hierarchy and bureaucratic roles (Frenken et al., 2020; Meijerink et al., 2021b) (Table 1). Like all profit-making organizations operating in market environments, OLPs are inherently embedded in market logic (Frenken et al., 2020). The complexity of these two logics emerges from how workers realize OLPs' operating environments. OLPs implement parallel market logic in which they compete in markets with other companies for market share by creating their own marketplaces for labor exchanges (Frenken et al., 2020; Meijerink et al., 2021b).

**Table 1:** Ideals of institutional orders (adapted from Thornton et al., 2012).

**Categories**	**Market logic**	**Corporation logic**
**Source of identity**	Faceless	Bureaucratic roles
**Basis of norms**	Self-interest	Employment in firm
**Basis of attention**	Status in market	Status in hierarchy
**Control mechanism**	Industry analyses	Organization culture

Studies have revealed that the complexity of market and corporation logic arises from the HRM activities of OLPs (Keegan and Meijerink, 2021; Meijerink et al., 2021b). The earlier described five governance principles have major unities with these HRM activities. OLPs are embedded in corporation logic when they manage access to OLPs (1. membership), job design, work allocation, compensations, instructions (2. rules), supervision, data collection (3. monitoring), performance appraisals and sanctions (4. sanctions), and positions of workers (5. hierarchies). Market logic attachment can be seen in activities in which OLPs provide workers with individual freedom and autonomy, like when they offer easy access to the platform environment, the possibility to choose when to work and for whom, the possibility to decline tasks, and by emphasizing self-employment and a lack of HRM activities (Keegan and Meijerink, 2021; Meijerink et al., 2021b). Governance generally refers to ways to decide these principles, but HRM refers to companies' direct activities toward workers. Thus, governance principles offer a framework through which HRM activities will be viewed in this study.

In addition to governance principles, the employment relationship will also affect this classification, suggesting whether a worker operates on OLP as an employee or an entrepreneur. As was mentioned, employment is the authentication of membership and shared agreement of company rules, and it covers many dimensions of governance principles. Interaction, autonomy, and power relations between OLPs and workers change considerably based on whether workers operate as self-employed or employed (Pichault and McKeown, 2019). Employment has also often been a central question when analyzing OLPs' legitimacy and can reflect the centrality of corporation logic (Keegan and Meijerink, 2021). Therefore, it is justified to analyze HRM and how it relates to employment or entrepreneurship. The next chapter will present the research question, data, and methodology of this research and how the classification was implemented using the described theories.

## Materials and methods

The article aims to answer the following question: *What models do online labor platform companies' HRM activities display?* The first step was to define what is considered OLP in this study and what OLP companies should be selected as research subjects.

### Data

The criteria for OLPs were formed based on the definitions of Eurofound and the ILO. All the others were introduced to research and typifications (e.g., Kuhn and Maleki, 2017; Kenney et al., 2020; Kovalainen et al., 2020; Keegan and Meijerink, 2021). The formed criteria of selected OLPs are summed up to four points:

1. There are at least three independent parties involved: the platform company (OLP), the client, and the independent work provider (in this paper, called “worker”).
2. The OLP has a digital platform for mediating tasks, projects, or fixed-term employment relationships.
3. The OLP positions itself as an intermediary between clients and workers.
4. The mediated service is labor.

The studied OLPs were found with the help of a search engine by finding companies' webpages or *via* articles or news using the research literature vocabulary. I also received suggestions from working life researchers and others stakeholders of OLPs. The first sample of OLP companies consisted of 39 cases and was collected in 2021. In 2022, the number of cases was supplemented to 46 with seven additional cases. The cases included two platform cooperatives to increase the coverage of the sample (Supplementary material). Classification is solely based on these OLPs and does not rule out possible models that could be recognized with an even wider sample.

Data consist of webpage texts and service terms for all OLP users or workers registering with OLPs. All textual material is from the webpages of OLPs and is thus publicly available. Website texts, such as operations presentations, frequently asked questions, and joining instructions, are mainly for marketing purposes. The quality and amount of text on web pages depend on OLPs and how they present themselves. It should be noted that sites used for marketing purposes can give a biased image of operations, despite not being false. Usually, they gave a quite compact overall image.

The second datatype, “terms of service,” is an agreement that expresses the rights and obligations of OLP users. They specify the nature of the legal relationship among parties involved in OLPs. Some OLP companies' terms of service are targeted to all users, but some also have their own terms for different user groups like service providers (workers) and clients. Terms of service identify the roles and obligations of each party, but they do not always open work mediation processes directly; they do it indirectly by framing parties' roles and permitted and unauthorized functions in work intermediation. A large part of the terms of service consists of privacy and data protection policies, which were not the focus of this analysis and were thus removed from the final data.

The data consisting of terms of service and webpage presentations cannot tell the operation practices; they can only tell how OLPs display them and the legal frameworks within which the work takes place. Data reveal the formal aspects of OLPs, but some functions, like how OLPs balance supply and demand in their markets and guide workers' actions and behavior with “soft control” techniques like incentives (e.g., Dieuaide and Azaïs, 2020; Connelly et al., 2021; Keegan and Meijerink, 2021), may differ in real life because they only appear while working. Because OLP companies themselves solely produce the material, there is also a risk that companies aim to hide some of their most controversial HRM activities. However, the data have also brought out management activities that were questioned in earlier studies, like sanctions based on only client reviews. Their usage may imply that they are expressed in OLPs' terms of service.

The data were found to be appropriate for outlining and finding the main characteristics of HRM activities and their emergence in digital work intermediation. While it is noted that the data cannot necessarily achieve all the HRM activities OLP companies have in their operations, the data were found to be sufficient to describe the ensemble of the work intermediation and working processes and operations that OLPs manage and the ones they do not. For example, while data cannot necessarily reach all the soft control techniques, they reveal if OLPs have rules and protocols for work intermediation and working. This determines the operations that OLPs allow themselves to manage and supervise. Data provide sufficient information on the extensiveness of OLPs' management and thus allow us to analyze the salience of market and corporation logic. The model descriptions in the result section have been slightly complemented with examples from earlier studies. The final research material consists of 99 documents from 46 companies. The material is in either Finnish or English.

### Methodology

Every OLP company in this research is its own case. Its data weres analyzed with a theory-guided content analysis method in which earlier classifications and studies on platform work guided the thematic basis of coding (e.g., Hsieh and Shannon, 2005; Schreier, 2012). Texts were analyzed and coded with the Atlas.ti software. The analysis phase consisted of multiple coding rounds, and the first ones were without guiding theories but relied extensively on earlier literature on OLPs. The first coding rounds found HRM activities that made distinctions between OLPs. In later stages, I included five governance principles into the analysis to assort and distinguish these activities more systematically. I classified OLPs based on shared qualities in governance principles, including forms of employment. In this paper, formed classes or categories will be called “models” by following conventional content analysis, aiming to create descriptive concepts and models of research phenomena (Elo et al., 2014). The word also better conceptualizes the nature of these models as different approaches and configurations to HRM rather than mutually exclusive categories. The classification consists of six HRM models. In the last phase of the analysis, I reviewed if the same model OLPs had shared target groups of workers.

In the analysis, I used a pattern-matching technique by Reay and Jones (2015). With this technique, governance principles and corresponding HRM activities were found and compared to characteristics of institutional logic ideals representing the patterns in this method. The object of the analysis was to find principles from the data that matched pre-created ideal types, which in this case were market and corporation logic ideals by Thornton et al. (2012). The benefit of the technique is that it enables us to recognize essential categories that would otherwise remain detached findings and to compare them to common reference points. The technique enabled us to make conclusions on the central logic behind the use or non-use of single HRM activities, which expressed the salience of market and corporation logic in all OLP cases and provided the possibility to make models on similar OLPs (e.g., Besharov and Smith, 2014; Reay and Jones, 2015). In the following sections, I will introduce how governance principles were found from the data and were analyzed in relation to institutional logic.

#### Membership

The first principle, membership, was analyzed regarding who has access to OLPs as workers and on what terms. This expressed how open OLPs are for workers to join, to what extent they manage workforce supply, and whether they represent more open marketplaces or closed organization environments.

#### Rules

In this study, rules that apply to work are divided into two groups based on whether they apply to the phase of work mediation or work processes. Work mediation is a phase where clients and workers meet *via* OLP and possibly negotiate the terms of work. Mediation rules define permitted and forbidden activities in negotiations between clients and workers or if OLPs manage task allocation completely without either clients' or workers' contribution (e.g., Maffie, 2020). The rules of work mediation indicate whether OLPs promote market-type interaction between clients and workers or, to what extent, they manage interactions and take responsibility for work allocation. Work processes are referred to here when talking about actual work performance, such as driving, repairing, and translating. Work process rules are direct instructions to workers, like working times, routes, or quality standards (e.g., Kuhn and Maleki, 2017; Krzywdzinski and Gerber, 2022). Work process rules express whether OLPs manage work performances and implementations of work and thus take responsibility for produced services in addition to labor intermediation.

#### Monitoring

The third principle, work monitoring, was approached by reviewing whether work processes or mediations are monitored by OLPs and how. This again revealed if OLPs took the authority to manage workers' actions or avoided monitoring and thus took a more marketplace-type position by transferring responsibility to clients. Analysis of this element also revealed the methods OLPs use for monitoring. They can be, for example, activities built into algorithms like ratings or tools to monitor work progress.

#### Sanctions

All OLPs held the right to exclude workers if they acted against the code of conduct or caused harm to the OLPs. The sanction principle was analyzed by whether sanctions applied to work processes or mediation monitored by OLP companies. This revealed whether detected failures or omissions impact workers' possibilities to operate on OLPs. Sanctions were considered corporation logic actions if they affected work allocation or workers' positions in OLPs' hierarchies and thus workers' possibilities to operate.

Rating schemes are involved in many OLPs but have different roles in operations and, thus, in HRM activities. In some OLPs, ratings are tools to guide and supervise workers and their performances. In others, they are references in workers' competence profiles. Ratings can also determine workers' positions in OLPs' inner rankings and affect their possibilities to get new tasks (e.g., Kahancová et al., 2020; Krzywdzinski and Gerber, 2022). Thus, ratings can be used to support market logic to promote workers' self-marketing or corporation logic when utilized for monitoring, sanctioning, or creating hierarchies. Therefore, ratings could be analyzed based on either logic or logic-intended use.

#### Hierarchies

The fifth principle, hierarchy, can be understood as either formal ones, which indicates stable orders of organization members, or informal ones, which emerges in social interactions between organization members (Diefenbach and Sillince, 2011). Since this research cannot access all the practical aspects of OLPs, hierarchies will only be analyzed in terms of how they appear in the data. In a formal hierarchy, all organization members' official roles and positions are clearly defined and demarcated from each other (Diefenbach and Sillince, 2011). The role of hierarchies is to produce predictable and uniform outcomes. While they can refer to top-down commands and control, hierarchies can also be seen in well-defined, ordered, and controlled tasks that aim for predictable results. This expresses that companies have internal authorities that define the direction and boundaries of operations (Spinuzzi, 2015).

If OLPs have rules for operations that lead to predictable and well-defined services, they may refer to hierarchical structures in companies and corporate logic. Another hierarchy-indicating factor is that OLPs have written hierarchical positions of workers, one indicator for which is the employment relationship. Hierarchy is not a synonym for corporation logic, but as the corporation logic ideal refers to traditional organization forms built on bureaucratic roles and statuses, any hierarchy-referring elements, like employment relations, can be considered evidence of corporation logic (Table 1). If OLPs have no expressed hierarchical positions like formal employment, or activities that restrict the freedom and work of independent workers, this refers to market logic.

Before proceeding to the results, it must be emphasized that no OLPs had HRM activities similar to each other. This required me to analyze the number and quality of similarities between OLPs when forming models. For example, while in many same-model OLPs, the work mediation activities may be displayed similarly, membership requirements may vary. I decided to emphasize HRM activities in work processes and work mediations in this classification to reveal better the possible power asymmetries and elements that may affect workers' operating and working conditions, autonomy, possibilities to gain work and income, and possibilities to compete and sell their services. Another noteworthy thing is that these models do not necessarily exclude each other. The same OLPs may have different digital platforms for different services, or there can be different services on the same platforms. Therefore, different models can operate simultaneously in the same OLP ecosystems.

## Results

Table 2 shows the names and short descriptions of each model according to their five governance principles and employment relations. Short explanations are under each principle. On the right side of the table are the summarized target groups of workers to whom these OLPs are marketed.

**Table 2:** OLPs' human resource management (HRM) models.

		**Governance principles**						
	**Models (46 OLPs)**	**Memberships**	**Rules**	**Monitoring**	**Sanctions**	**Hierarchy**	**Form of employment**	**Target groups of workers**
1	Free marketplaces (9 OLPs)	Requirements for memberships vary from completely open to those limited only to confirmed professional	Rules of work between clients and workers. Work mediation slightly managed	No work monitoring	No written sanction practices	Open marketplaces with no hierarchies	Entrepreneurship/self-employment	Skilled workers for marketing and selling their services
2	Managed marketplaces (12)	Requirements for membership vary from completely open to those limited only to confirmed professional	Rules of work between clients and workers. Work mediation managed by OLPs	Work mediation monitored by OLPs	Sanctions for rule breaches in work mediation	Open marketplaces with no hierarchies	Entrepreneurship/self-employment	Skilled workers for marketing and selling their services
3	Temporary employment platforms (6)	Membership either completely open or through interviews with OLP representatives	Rules of work determined by clients Work mediation managed by OLPs	Work mediation monitored by OLPs	No written sanction practices	Open marketplaces. OLPs as employers of workers	Temporary employment/entrepreneurship/self-employment	Uneducated, students or graduates. People looking entry into working life
4	Managed networks (5)	Requirements for membership vary from completely open to those limited only to confirmed professional	Rules of work negotiated between parties Work mediation managed by OLPs.	Work mediation monitored by OLPs	No written sanction practices	OLPs as networks of experts. OLPs have power on who will receive assignments	Entrepreneurship/self-employment	Experts from different fields and positions
5	Managed work processes (10)	Only minor requirements for memberships, such as drivers' license. Almost all OLPs decide who can get membership	Rules of work defined by OLPs Work mediation managed by OLPs.	Work processes and mediation monitored by OLPs	Varying sanction practices for weak work performances and non-compliances	OLPs as facilitators of tasks Well-defined tasks	Entrepreneurship/self-employment	Underemployed, students, migrants. People seeking extra income
6	Managed like corporations (4)	High requirements such as confirmations of competence and experience, and interviews with OLP representatives	Rules of work defined by OLPs Work mediation managed by OLPs	Work processes and mediation monitored by OLPs	No written sanction practices	OLPs responsible for work and workers. Well-defined tasks	Employment/entrepreneurship/self-employment	Professionals, people seeking flexible working opportunities

### Free marketplaces

A characteristic of the first model is that OLPs are, for the most part, free to operate for workers. Free-market OLPs have only minor or no control over work mediation and do not manage work processes. The terms of work are directly negotiated between clients and workers. Membership requirements vary between OLPs. Two require a certain educational level, and one checks the backgrounds of new workers, but most studied OLPs had no formal access requirements. The model has OLPs from a variety of fields, e.g., construction, marketing, and IT services, but almost all represent professional work. Promoting workers' businesses and competence is at the center, which positions these OLPs as marketplaces.

Free marketplace OLPs allow clients to leave invitations to tenders or browse the selection of potential and available workers. Clients and workers make agreements to meet *via* OLPs and to the terms of the work. OLPs do not manage these negotiations, but they offer means of communication and payment arrangements. Overall, these OLPs have very little of anything that restricts users' activities. They mostly rely on user trustworthiness instead of extensive monitoring. “*We only provide a forum via our website for users to connect. We do not take part in any contractual arrangements between users” [Terms of Service, GigExchange]*.

Most of these model OLPs use rating schemes in which clients can rate workers and, in some cases, workers can evaluate clients too. This rating is often publicly available in workers' competence profiles and directly affects workers' competitiveness in the OLP marketplace. Compared to market and corporation logic ideals, these OLPs do not have hierarchical roles but rather trust the disciplinary power of labor markets (e.g., Vallas and Schor, 2020). Workers operate as self-employed individuals, and OLPs are marketed as places to advertise and manage their services, acquire client bases, and employ themselves on their own terms.

### Managed marketplaces

The second model consists of OLPs with a similar idea to the previous one: marketplaces and business management tools for self-employed workers. The difference is that in these, the work mediation phase is more managed by OLPs. Like in the first model, access requirements vary between OLPs; some require work samples as proof of experience, some arrange pre-interviews, and some are open to all. In addition to differences in work mediation management, the managed marketplace model includes more international online freelance platforms (e.g., International Labour Organization, 2021) and companies operating in various countries than the free marketplace model.

These OLPs do not have control over the work itself, with a couple of exceptions, in which they collect data on work performances and working hours for payment arrangements. This data and earlier studies also reveal that some of these OLPs can offer clients tools to supervise work performances, for example, by having timers for working and systems that take intermittent screenshots from workers' computers (Seppänen et al., 2021). Most commonly, these OLPs manage only mediation with varying activities, such as managing communications between parties, or by having restrictive rules for collaborations, such as limitations on canceling gigs. Many of them also use algorithms and different types of memberships to determine the visibility of workers' profiles (e.g., Maffie, 2020).

Workers are given sanctions when they fail to notify OLPs on not meeting made task requirements or for canceling the tasks in the late minute. They can be monetary or involve exclusion from OLPs. A couple of the OLPs also exclude or downgrade workers if they do not manage to offer promised services to clients or do not reach a defined quality level. This often occurs if workers get too many bad reviews or low client ratings. “*If the [worker] receives at least three bad reviews for the service within 6 months, the service provider has the right to cancel the contract immediately. This indicates that the [worker] is incapable of offering high-quality products and/or services to the users” [Originally in Finnish] [Terms of Service, Urakkamaailma]*.

Rules, monitoring, and sanctions all express that OLPs following this model take responsibility for providing quality and reliability to clients without being direct service providers. Workers compete in OLPs with their own services and can choose their tasks and define their prices. Workers are directly responsible for clients, but in some cases, OLPs' functionalities, like ratings or other gained approvals, may create certain rankings in these marketplaces. These rankings directly impact workers' competitive positions and the visibility of their profiles. The model has OLPs from many different industries that place emphasis on high-skill professional work. Therefore, OLPs are marketed to experts from different fields.

### Temporary employment platforms

Nominal for this Temporary employment platforms is that, in addition to being marketplaces like previous models, OLPs are also employers of workers. Some allow membership without any restrictions, while others have pre-interviews for applicants. In all cases, membership is advertised as not requiring extensive experience or expertise. OLPs do not define the rules or contents of tasks, but often they certify workers and ensure that clients will get a suitable workforce, for example, if tasks require certain qualifications or licenses. Almost all of these OLPs have rating systems, most of which are reciprocal; thus, both clients and workers can review each other. Ratings are used as insurance for parties' reliability and marketing workers' competence.

Many of the functionalities resemble the above-described marketplace models; thus, market logic appears as the more central logic in interactions between clients and workers. Workers are free to bid on themselves for tasks or projects published by clients. Clients are also free to contact and choose workers from OLPs. When work has been agreed upon, an OLP company or external staffing company forms an employment relationship with the worker for the task's duration in accordance with the framework agreement. During the working time, the OLP company or the staffing company is the worker's employer, which refers to the direction of corporation logic. Some of these OLPs also offer the possibility for workers to operate as self-employed individuals or to form employment relations directly with client companies. Some OLPs in this model are owned and run by staffing companies, which may express that this form of platform work can represent a platformization of traditional staffing companies (Leiponen and Kotiranta, 2020), or OLPs are adopting flexible models of employment into their operations.

Most of these OLPs are intended for workers looking for access to working life. Especially OLPs without major requirements for membership market themselves to those in the early phases of their work careers. These OLPs are marketed as ways to gain work experience through short assignments and meet employers easily. Two of these are mainly for educated people in their fields, but the same “stepping stone” idea is visible on their websites as they market themselves as pathways to full-time labor or more stable income. “*[OLP] was born out of the desire to tear down needless barriers to job opportunities and give as many people as possible the chance of getting valuable work experience” [Webpages, WorkPilots]*.

### Managed networks

The model of managed networks includes OLPs that could be seen as very different from each other based on their sectors and user groups. They gather confirmed experts from fields like cleantech, social media marketing, and construction. These OLPs are designed to mediate complex tasks that necessitate specialized skillsets and in-depth knowledge, in which workers' suitability is unequivocally hard to identify by clients without the knowledge of work and industry-specific factors. That may be why OLPs manage work mediation more than previous models.

Access criteria vary between these OLPs; however, OLPs frequently validate workers' competency in certain phases. This happens either in the access phase to OLPs or when workers apply for tasks or projects *via* OLPs. Despite the diversity of OLPs, they manage work mediation in two ways. In the first, the client defines the content of the work and uses an OLP independently to find a suitable worker for the assignment. In another, clients, together with OLPs' own experts, create an assignment or a project for which they select a suitable workforce from the platform network. Either OLPs allow clients to directly utilize these networks themselves, or OLPs serve more as consultants or agencies and participate in finding suitable workers to meet clients' needs from their networks. Some OLPs allow both described ways.

OLPs can extensively manage work mediation and take responsibility for clients getting a quality workforce. Workers who have received access to the networks can freely apply to assignments provided by clients and price and advertise themselves, but very often, OLPs have power over who will get assignments or who will be presented to clients. Evaluation and selection of workers are based on either algorithms, expert reviews, or both. OLPs usually do not manage work performances. The allocation of work and meeting of clients and workers is sometimes monitored and managed by following and limiting messaging and assignment details before an agreement. Some OLPs have sanctions for the omission of rules. In one OLP case, workers are obliged to announce the assignments agreed upon outside the OLP. Omission of this leads to exclusion from OLP. One OLP sanctions if they do not fulfill the minimum requirements of work determined by OLP company.

In comparison to previously presented models, operations differ considerably. This model is a bit complicated in terms of market and corporate logic. OLPs' managerial power over workers may create a certain hierarchy inside these networks. Workers do compete and advertise their businesses, but they do not compete directly for clients but also for the approval of the OLPs, which is why OLPs cannot be considered marketplaces only. OLPs cannot be attributed completely to corporation logic either because they have no hierarchical positions, rules for work, or demands for workers. All these OLPs are marketed to experts to help them find work that matches their competence. Workers can be company representatives, independent consultants, or casually working freelancers. Besides work intermediation, some of these OLPs are marketed as networks to create contacts with other experts and clients in the field.

### Managed work processes

This model consists of OLPs, all of which operate in the transportation sector, offering either taxi or delivery services. They often have high competence requirements for memberships as workers. OLPs offering delivery services require a smartphone, bicycle, or car, as well as national work permits, which indicates that one generic worker group for them is migrants. OLPs offering taxi services also require legal transport permits. The difference compared to earlier described models is that these OLPs mediate short assignments instead of projects or client-determined tasks. Many of the companies have global operations, and their volume and number of platform workers are high.

Nominal for this model is OLPs' management over work processes. OLPs serve as distributors of tasks, meaning that they decide the workers to whom tasks are offered. Task allocation is guided algorithmically based on distances, workers' availability, and other OLP- and work-specific factors. In many cases, rules extend to work processes, including time limits for work, pre-calculated routes, defined rules for work performance, and, in some cases, rules for behavior. The contents of the tasks are pre-defined, and workers must act within the given framework and compensations.

On some managed work processes OLPs, tasks are monitored by collecting data *via* mobile applications. This data includes working times, speed, routes, and communications between clients and workers. Often, OLPs also provide clients with tools to monitor transportation and their progress. What has to be underlined is that all these OLP cases manage work processes with some of these methods but in very different ways and intensities. Some OLP companies' rules are also more coercive than others. “*The [worker]'s obligations: Delivery of the [product] specified in the Task to subscribers by 6:30 a.m. on weekdays, 7:00 a.m. on Saturdays, and 7:30 a.m. on Sundays in accordance with separate instructions in an area selected by the [worker], which is also called a delivery district” [Terms of Service, EarlyBird]*. Sanctions in some OLPs are affiliated with data collection on workers' performances. How sanctions affect workers' possibilities to receive new tasks is not always clearly expressed and can differ between OLPs. In one case, non-compliance with the rules may lead to losses in compensation. In another delivery OLP, poor performance will lead to a decline in the internal rating, which affects workers' possibilities for booking new shifts.

More than half of these OLPs have rating schemes for clients to evaluate workers; in some cases, workers can also evaluate clients. This rating has different effects on workers. On a couple of taxi service OLPs, the rating impacts drivers' rankings and, thus, the possibilities of getting new task offers from OLPs. An average rating below a certain level will lead to exclusion from OLPs. “*We track the quality of our service using customer feedback. Drivers with high ratings get orders first, and drivers below the minimum threshold are automatically blocked from receiving orders” [Webpages, Yango]*.

In terms of hierarchy, these models have certain power relationships. OLPs decide rules, pricing, and sanctions and manage work distribution. Rather than managing only markets, they often also manage processes, outputs, and work quality. Workers do not directly compete for clients but for positions on the OLPs and their algorithms. However, formal employment is not involved in these models. These OLP companies emphasize that workers are free to use the OLPs when suitable for them and are not obligated to work. Based on earlier studies, many of these companies have HRM activities to direct and encourage workers to work at certain times and locations (Dieuaide and Azaïs, 2020; Schor et al., 2020). OLPs advertise themselves as places for the underemployed or students to gain extra work and income. The ease of access and flexible earning opportunities are marketed to support situations where full-time work is not necessarily desirable or possible.

### Managed like corporations

The last model consists of OLPs, whose defining elements are strict membership requirements and strictly managed work tasks. In many ways, these OLPs are reminiscent of traditional working arrangements, with their rules of procedure and workers' positions. An employment relationship is possible for most OLPs, but there are also self-employed workers in all these cases. As mentioned, access to these OLPs is often difficult and requires formal competence. Most of the case-OLPs operate in the field of translation or interpretation. All of them arrange interviews with applicants before accepting them on their platforms. Like in the previous model, these OLPs mostly mediate short assignments, whose contents they standardize and manage.

All of the model's OLPs take responsibility for the quality of their work. They have strictly defined rules for meeting the quality standards of their services. They also define the prices of services and allocate tasks to appropriate workers according to qualifications, availability, and work content. All OLPs are responsible for work, and workers can produce the required quality. Quality is monitored by collecting data on work performances, evaluating results, and collecting ratings from clients. In one case, the rating will affect opportunities to work on the OLP, but the others had no sanctioned practices in their terms of service. “*The service records, e.g., the worker, the user, and the duration and time of [work] performed through the service for purposes of quality control, invoicing, and analytics [Terms of Service, Tulka]*.

These OLPs often highlight their work community features, like the active role of workers in developing services and regular development discussions with managers. Many of them advertise training and self-development opportunities and flexible work possibilities at different times and locations. All these imply that OLPs position themselves more as employers than purely as intermediaries or marketplaces. As indicated previously, in most of these OLPs, there are both self-employed and employed workers. This may indicate that employment is for different services than self-employment, that working as a self-employed individual is a trial period before employment, or that the status is to be decided by the workers.

OLPs of this model are advertised to those searching for flexible work opportunities with competitive remuneration and benefits. They are often marketed as multilingual because the emphasis is on the translation sector. The membership threshold is high, as seen on their websites, where the OLPs underline on exact interviews and hard-filter workers with high competence. While there was no clear industry or work emphasis in the first four models, the emphasis is quite visible in this and the previous model. It can relate to the nature of work and the possibilities for formulating it into standardized assignments. The difference from the previous model is the closer connection between OLP companies and workers through employment and other company activities.

## Discussion and conclusion

The research on organizations' responses to the complexity of various institutional logics has gained wide interest. It has proven to be useful in describing institutional changes and pressures in various fields and organizational environments. It is not only relevant in cases of OLP companies but everywhere where organizations face intersecting values, identities, and expectations from organizations' internal or external actors (e.g., Greenwood et al., 2011; Raynard, 2016; Vermeulen et al., 2016). In the case of OLPs, the complexity of market and corporation logic is found to be effective in describing contradictions between HRM activities and the operational freedom of platform workers (Keegan and Meijerink, 2021). However, just like in other organizations, these are not the only institutional logics that modify OLPs. Earlier literature has suggested that many OLPs face demands from professional, state, and community logic (Frenken et al., 2020; Keegan and Meijerink, 2021). Considering the models of this research, professional logic may appear as a major affecting force in a managed network (4) and managed like corporation models (6). Further studies will expand the scope of institutional logic analysis to reveal other influential forces behind OLPs' HRM activities.

This classification advances and enriches studies on OLPs' institutional logic complexities presented by Frenken et al. (2020) and Keegan and Meijerink (2021). The results indicate that the complexity of market and corporation logic and the salience of this complexity can be evaluated and classified by reviewing the HRM activities of OLPs through governance principles. What this study has brought out is that OLPs have a different extent of HRM activities due to different governance principles. While some OLPs manage work processes without having wider restrictions on who can utilize OLPs for working (Model 5), others may manage the entrance phase but allow more freedom for workers in negotiations with clients and work (Models 1, 2, and 4). Solutions to manage selected governance principles to reflect that OLP companies are partial organizations (e.g., Ahrne and Brunsson, 2011), and this “partiality” gets widely different implementations.

The complexity of different logics is not different from the historical development of organizational control, which has always been considered to be constituted by varieties of different techniques adapted into organizational practices in strategically different ways and at different times (Hyman, 1987; Ivanova et al., 2018). Technology adoption also depends on work characteristics, uncertainty, and competition in companies' external environments, such as the market environment (Beer and Mulder, 2020). The coordination of work by using technology as an endeavor to manage market competition has already been expressed in Coase's (1937) article “The nature of the firm,” which claimed that inefficiently functioning markets, a technological development that will decrease transaction costs, make companies less vertically structured and less interested in committing workers through an employment relationship. With this in mind, the whole phenomenon of platform work and differently structured OLPs may appear as a continuum of this same technical development responding to the uncertainty of global and local market environments.

This classification is a snapshot of the time and situation of constantly evolving digital work intermediation. Because of the Institutional logic complexities, some OLP companies' legality has been questioned, and they have caused worker mistreatment with their management activities. Thus, OLPs are facing different amounts of public and administrative pressure to modify their models. This paper is written when characteristics of employment relations and rules for algorithmic management in platform work are being outlined (e.g., European commission, 2021; Hießl, 2022). Revelations have also been made about Uber's large-scale lobbying campaigns that have managed to make changes national legislations in many countries (Davies et al., 2022). OLPs' institutional environment is constantly changing, which is why created models should not be considered static and definite, as they will most definitely change over time and need updates and refinements. Some OLP companies have removed HRM activities from their operations to avoid being considered employers (Interviews with OLP leaders, 2021). At the same time, OLPs have taken on or have been forced to assume employer responsibilities (Hießl, 2022). It can also be that OLPs adopt strategies from other OLP companies, and thus the question of what types of OLP models will gain popularity and institutional legitimacy will be revealed over time.

Another reason why models should not be considered static is that they are not mutually exclusive. Some of the studied OLPs had features from different models, and in some cases, multiple platforms in the same OLP ecosystem worked with different models. Multinational freelancer OLPs Upwork and Fiverr have different services for different memberships or “seller levels.” Even though defined here as managed marketplaces, these different memberships and their services modify the nature of the intermediation in a way that some of their models resemble both temporary employment platforms (Model 3) and managed networks (Model 4).

There were also two platform cooperatives included in this sample. One represented the free marketplace model (1), and the other the model of managed work processes (5). The latter was challenging to classify because, even though there were written rules for work performance and the OLP handled the allocation and work-related matters with algorithmic procedures, the development of services was user driven. No sanctioning practices or other elements created hierarchies, so the power imbalance question differs from OLPs in the same model. The power imbalance question also differs because ownership relates to power distribution and its centralization or decentralization in platform ecosystems (Hein et al., 2020). Therefore, the models presented here will require more research on labor platform cooperatives and their institutional logic.

The findings underline the flexibility and modularity of digital platform ecosystems and that, in some cases, these models overlap with each other. However, this does not scatter the formed models but only reveals that models are not necessarily direct descriptions of what OLP companies are like or what classes they belong to. Rather, they describe how HRM activities and interrelationships between OLPs and workers can be arranged. Models should be viewed as indicative, general, and highlighting important elements of HRM but should not be used to make direct inferences about OLPs' legality or power asymmetries. However, this classification may help to make important elements of OLPs' HRM visible by developing concepts to describe the working environments that differently managed OLPs create.

For example, different strategies for work allocation help decide workers' decision power and what kinds of needs OLPs can be utilized. Let us take self-marketing as an example. In marketplace OLPs (Models 1 and 2), the decision-making power on marketing is with the workers. In managed networks (Model 4), this power is limited by OLPs. In managed work processes (Model 5) and managed corporations (Model 6), the possibility of workers marketing themselves is minimal or non-existent. Therefore, OLPs of the latter models are not particularly suitable for service marketing but could be utilized to acquire pre-defined tasks for direct income needs. Recognition of what kind of working environments and opportunities for operations these HRM activities create will enable us to analyze the possibilities and issues associated with different models. This helps to recognize the elements affecting workers' experiences, wellbeing, and fairness perceptions in different OLPs. In addition to HRM activities' great impact on worker experiences, these activities can also affect what workers expect from OLPs. Workers entering marketplace OLPs (Models 1 and 2) may seek only additional forums to market their businesses, whereas people entering managed work processes OLPs (Model 5) may expect continuous task offers and definite income, and thus more responsibility from OLP companies.

Earlier classifications have separated OLPs based on skill levels, sectors, and working methods (e.g., Eurofound, 2018; Howcroft and Bergwall-Kåreborn, 2018; International Labour Organization, 2021). Also, this classification shows some sectoral and skill-based similarities and differences between the models. Marketplace OLPs (Models 1 and 2) and managed networks (Model 4) emphasize experienced professional workers, whereas temporary employment OLPs (Model 3) aim to offer possibilities mostly for those in the early phases of their work careers or experts who do not have extensive experience yet.

Sectoral connections are most visible in managed work processes (Model 5) and managed like corporations-models (Model 6), which consist mostly of transportation and delivery (Model 5), and translation (6) sector OLPs. This may indicate that certain HRM models have a more legitimized position within sectors. OLPs with more intensive HRM also advertise working opportunities for those in a more vulnerable position in labor markets, like migrants or the uneducated. On the other hand, OLPs with less intensive HRM offers possibilities for the educated and those generally in a more stable position. This indicates that OLPs not only develop in different directions but also that their working opportunities are targeted at different people, treat them differently, and offer them different levels of autonomy.

As previously stated, earlier studies have focused more on OLPs that have caused contradictions with misclassifications of entrepreneurs and algorithmic management activities. In light of this classification, focusing research on OLPs with intensive HRM will give a one-sided picture of OLPs' HRM activities. The results indicate that OLPs strategically use different algorithmic or non-digital HRM activities for different governance principles. There are also sectoral, task, and work-related factors that may affect OLPs and the extent of their HRM activities.

Models can add value to the research on OLPs' societal impacts. Research on platform work precariousness has recognized versatile risks to income, job security, social security, autonomy, etc. (e.g., Kahancová et al., 2020; Krzywdzinski and Gerber, 2020; Schoukens, 2020). Models could help to understand and outline qualitative differences in precariousness for different types of platform work and identify OLPs' impacts on workers' autonomy and security. Taken together, the role of models is not to challenge earlier research and findings but rather to bring a new instrument to assess the challenges and opportunities of platform work already identified and deepen the analysis of them. Future research calls for more detailed ethnographic and user-based data and research on OLPs' HRM activities and logic complexities.

## Data availability statement

The original contributions presented in the study are included in the article/Supplementary material, further inquiries can be directed to the corresponding author.

## Author contributions

In terms of data collection, analysis, and writing, JI was the only author who contributed to the article.
