# Supplementary material for: HRM models of online labor platforms: Strategies of market and corporate logics
Source: Front Sociol. 2023 Jan 6;7:980301. doi: 10.3389/fsoc.2022.980301 (PMC9853187; doi:10.3389/fsoc.2022.980301)
Supplement: Supplementary file 1 [file Table_1.DOCX]

Supplementary Material

**Supplementary table 1:** Researched OLP companies and times of their data collection

| **OLP company** | **Time of the data collection** |
| --- | --- |
| Applicolis | 3/2022 |
| Babysits | 3/2022 |
| Bolt.Works | 4–5/2021 |
| Budbee | 3–5/2021 |
| Builderbot | 4–5/2021 |
| Cabo (ridesolutions oy) | 3–5/2021 |
| Care.com | 3/2022 |
| EarlyBird | 3–5/2021 |
| Extranet (extraajat oy) | 5/2021 |
| Ferovalo Oy | 3–5/2021 |
| Fiuge | 3–5/2021 |
| Fiverr | 3/2022 |
| Foodora (Delivery Hero Finland oy) | 3–5/2021 |
| Freelancer.com | 3/2022 |
| Freetalent | 3–5/2021 |
| Freska | 3–5/2021 |
| Gigexchange | 3/2022 |
| Gikker (Pocket Manager Finland Oy) | 3–5/2021 |
| Gixon | 5–7/2021 |
| Helpdor | 5/2021 |
| HR comcom | 3–5/2021 |
| Industryhack | 3–5/2021 |
| Kjelp | 3–5/2021 |
| Kodinplaza | 4–5/2021 |
| Kodinpro | 3–5/2021 |
| Lakiareena | 3–5/2021 |
| Minduu | 3–5/2021 |
| Muuttomaailma | 5/2021 |
| MyXline | 5/2021 |
| Nerot.fi | 3–5/2021 |
| Semantix | 4–5/2021 |
| Seure | 5–7/2021 |
| Solved | 3–5/2021 |
| Somessa.com | 4–5/2021 |
| Sumpli | 3–5/2021 |
| Superskills | 3–5/2021 |
| Transfluent | 4–5/2021 |
| Treamer | 3–5/2021 |
| Tulka | 3–5/2021 |
| Uber Finland oy | 3–5/2021 |
| Upwork | 3/2022 |
| Urakkamaailma | 6/2021 |
| Valokuvaajat.fi (polarico oy) | 3–5/2021 |
| Wolt | 3–5/2021 |
| Work Pilots | 3–9/2021 |
| Yango (Yandex Oy) | 5/2021 |
